# Extracellular vesicles isolation: a focus on magnetic beads-assisted platforms

**DOI:** 10.3389/fbioe.2025.1646385

**Published:** 2025-09-24

**Authors:** Xinghao Lan, Danyu Li, Yancong Yu, Sumedha Nitin Prabhu, Guozhen Liu

**Affiliations:** Integrated Devices and Intelligent Diagnosis (ID^2^) Laboratory, CUHK(SZ)-Boyalife Joint Laboratory for Regenerative Medicine Engineering, Biomedical Engineering Program, School of Medicine, The Chinese University of Hong Kong, Shenzhen, China

**Keywords:** extracellular vesicles, magnetic beads, evs isolation, magnetic beads synthesis, microfluidics, affinity-based isolation

## Abstract

Extracellular vesicles (EVs) play a crucial role in cellular communication and hold significant potential for diagnostic and therapeutic applications. However, the efficient isolation of EVs remains a challenge due to their heterogeneity and the presence of contaminating particles. Traditional isolation techniques, such as ultracentrifugation and size-exclusion chromatography, often result in low yield and purity. This review focuses on magnetic bead-assisted platforms as a promising approach to overcome these limitations. Magnetic beads (MBs) offer high specificity, reproducibility, and efficiency in EVs isolation, enhancing their utility in biomedical research and clinical applications. The synthesis and surface modification strategies of MBs are discussed. Additionally, advancements in microfluidic-integrated platforms and multiplex bead-based assays for EVs isolation are explored. The review highlights strategies to optimize EVs capture efficiency and outlines future directions for standardizing magnetic bead-based isolation protocols to improve their applicability in precision medicine.

## 1 Introduction

Extracellular vesicles (EVs), secreted by cells, are membrane-bound carriers of molecules like proteins, lipids, nucleic acids, and glycans, reflecting their parent cells’ health status. They are vital in precision medicine, serving as biomarkers for disease tracking and as therapeutic agents due to their ability to deliver effector molecules to recipient cells. Recently, there has been a growing interest in the role of EVs in disease progression. EVs contribute to disease progression, such as tumors, through various mechanisms, including angiogenesis, immune evasion, and alteration of the tumor microenvironment. The diagnostic potential of EVs has come into notice by their stability and accessibility in biological fluids, such as blood, urine, and saliva (Boukouris et al., 2015). EVs can also cross the blood-brain barrier (BBB), providing a unique opportunity to diagnose and treat central nervous system disorders. On the one hand, EVs have emerged as promising tools for the targeted delivery of drugs, genes, and other agents to treat CNS disorders. On the other hand, the biomolecules carried by EVs, such as proteins, RNA, and DNA, can be easily detected in peripheral blood.

**FIGURE 1:**
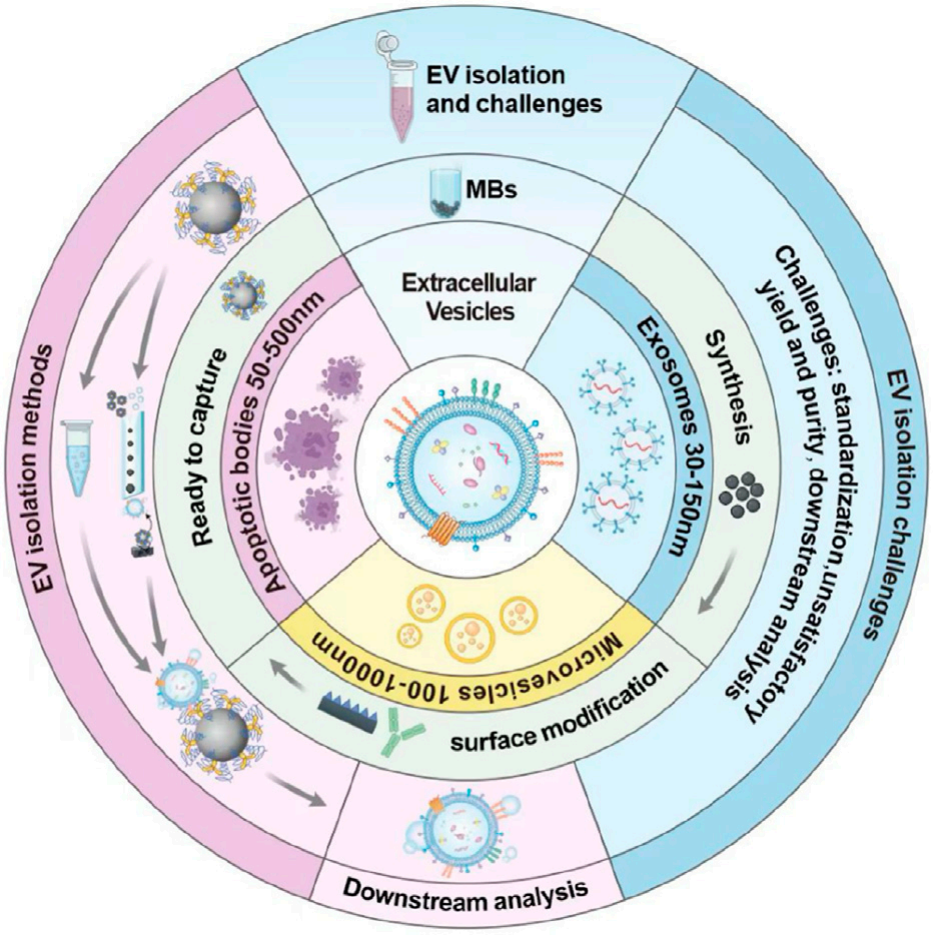
Schematics showing the contents of this review.

Classical EVs encompass exosomes, microvesicles, and apoptotic bodies, which have diverse origins. Few studies have described the separation and characterization of these EVs subgroups as their similar size and density characteristics. Therefore, this review collectively refers to all subgroups as EVs. Currently, there are still several challenges in the EVs isolation and identifying the source that need to be addressed, Some significant issues, including EVs heterogeneity, contaminating particles, and yield and purity, must be taken into consideration (De Sousa et al., 2023). EVs isolation methods including ultracentrifugation, size exclusion chromatography, and immunocapture have been widely used in EVs research (Bordanaba-Florit et al., 2021; de Menezes-Neto et al., 2015; Liangsupree et al., 2021). However, different isolation methods may lead to differences in the isolated EVs size distribution and cargo content. One of the main challenges in isolating EVs is their heterogeneous size, shape, and biomolecular composition, which significantly complicates the purification (Bordanaba-Florit et al., 2021). Isolation methods that rely on size-based separation, such as ultracentrifugation and size exclusion chromatography, may enrich a subset of EVs, leading to variable yields and specificity. Pre-analytical variables, such as sample collection and storage conditions, can also affect EVs isolation and characterization. Another challenge is the presence of contaminating particles in EVs preparations, such as lipoprotein particles and protein aggregates (Stranska et al., 2018). These particles can co-purify with EVs during isolation and interfere with downstream analyses, leading to misinterpretation of experimental results. Standardization of EVs isolation and methods for EVs characterization is critical for advancing our understanding of EVs biology and clinical relevance. The growing demand for EVs detection as critical biomarkers in clinical diagnostics and therapeutics shows the limitations of the conventional isolation methods, which often result in low purity, low efficiency, and long processing time. Unlike existing reviews *that broadly cover diverse EVs isolation techniques* (Shami-Shah et al., 2023; Zhao et al., 2021; Stam et al., 2021; Konoshenko et al., 2018), this review provides a focused and in-depth analysis of MB-based strategies, emphasizing their transformative role in EVs research. By centering on MB platforms, we systematically highlight their distinct advantages—including rapid processing, superior yield, exceptional purity. This review not only consolidates the latest advancements in bead-based technologies but also offers a critical evaluation of their potential to address key challenges in EVs isolation, such as scalability and reproducibility. This specialized perspective bridges a gap in the literature, serving as a valuable resource for researchers seeking efficient, standardized, and high-performance EVs isolation solutions. In this review, the synthesis methods and modification strategies of MBs are first introduced. Then, some EVs isolation strategies based on MBs are presented. Finally, the factors that may affect the EVs isolation efficiency are discussed and the measures that could increase the isolation efficiency are proposed. The basic workflow of the isolation processes is shown in Figure 1.

## 2 Different strategies for EVs isolation

The classical methods for isolating EVs and their comparison are shown in Table 1. More often now, methods with higher specificity or combinations are used to enhance EVs capture efficiency. Generally, the isolation and separation strategy for EVs is carried out in three main areas: size, charge, and affinity.

**TABLE 1:** A brief summary of conventional methods for isolating EVs.

Method	Description	Advantages	Disadvantages	Specific sample
Ultracentrifugation	EVs are separated by their sedimentation rate under high centrifugal force.	High yield and purity regarded as the gold standard (T et al., 2006)	Time-consuming and may lead to co-purification of non-vesicular components	Cell culture supernatants, blood plasma/serum, and urine
Size-exclusion chromatography	Separates EVs based on size using column filtration	Easy to use and can be automated; compatible with downstream applications	Low yield compared to other methods (de Menezes-Neto et al., 2015)	Plasma, serum, ascites fluid (Koliha et al., 2016)
Immunoprecipitation	Uses specific antibodies to isolate EVs bound to targeted proteins	Highly specific and sensitive; allows for the isolation of subpopulations of vesicles	Antibody-dependent, which may limit its application in some condition	Plasma, milk, saliva, and urine
Microfluidic-based separation	Fast, easy to use, scalable, suitable for large-volume samples	High throughput and accuracy; reliable isolation of specific EVs subpopulations (Harenberg et al., 2012)	Equipment costs and complexity may limit widespread adoption	Plasma and urine
Polymer-based precipitation	A polymer solution is added to sample, creating a cloudy mixture containing EVs which can be recovered through precipitation	Fast, easy to use, scalable, suitable for large-volume samples	Contamination with protein aggregates or other impurities may occur (Gardiner et al., 2014)	Plasma, urine, and saliva
Electrophoresis	This method can be used to purify specific subpopulations of EVs based on their electrophoretic properties	High resolution and specificity; can isolate EVs subpopulations (Long et al., 2013)	Requires specialized equipment; low yield compared to some other methods	Plasma, urine, and cerebrospinal fluid (Chiasserini et al., 2014)

### 2.1 Size-based techniques

**FIGURE 2:**
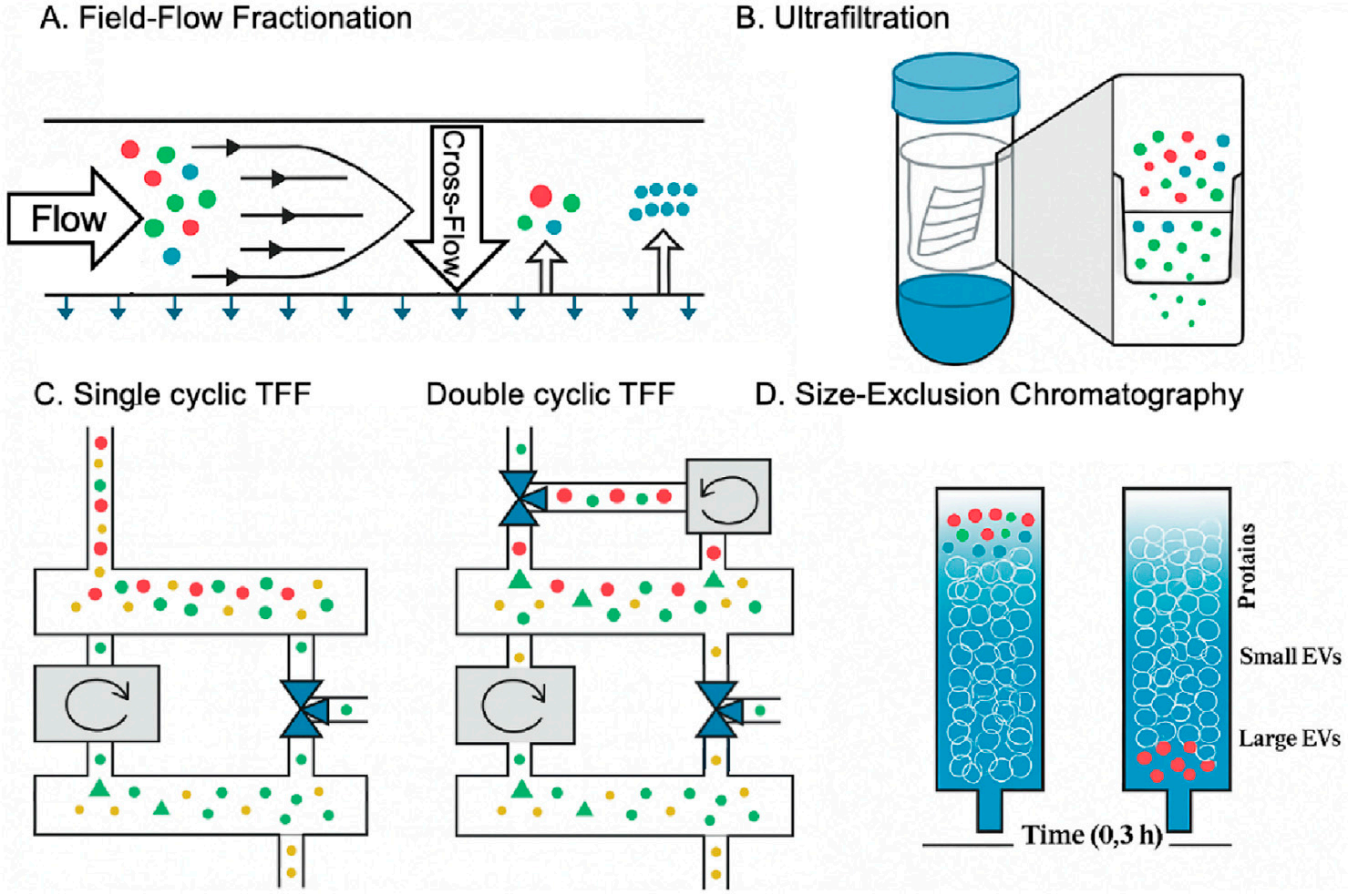
EVs biogenesis, subpopulations, and conventional and novel methods of EVs isolation based on the size (Kim et al., 2021; Amiri et al., 2022). **(A)** In the fractionation field, particles accumulate at different membrane positions depending on their size. Separation occurs when the diffusing and cross-flow forces are balanced. **(B)** Ultrafiltration separates particles by forcing them through a filter *via* centrifugation, with separation depending on the filter’s pore size. In contrast, polymer co-precipitation relies on steric exclusion, where PEG aggregates particles into clusters that can be easily pelleted through low-speed centrifugation. **(C)** A schematic representation demonstrates three distinct approaches for segregating fluorescent beads by size: (1) red particles (>200 nm), (2) green particles (30–200 nm), and (3) yellow particles (<30 nm. **(D)** Size exclusion chromatography, which separates particles based on size, is one of the most common methods for obtaining a large volume of exosomes due to the lack of protein contamination and the ability to purify the exosome on a large scale.

Size-based separations for EVs include chromatography and filtration (Figure 2). Size exclusion chromatography (SEC) is a chromatography-based method that has been used to fractionate molecules based on their hydrodynamic size or molecular size (Sorci and Belfort, 2014). SEC has a wide range of applications and can isolate EVs from various biofluid samples (Williams et al., 2023). It is a high-recovery method for isolating EVs based on size separation, although it has some limitations related to lack of specificity due to the presence of contaminants with similar size profiles (Lozano-Ramos et al., 2015). In addition, it can successfully remove serum albumin from serum samples, reducing the probability of protein contamination. However, other proteins in a similar size range to EVs, such as lipoproteins, will inevitably be co-isolated, which is a limitation of this method (Koster et al., 2021). Ultrafiltration (UF) is another popular separation technique based on size. It is a promising method for isolating EVs due to its fast and simple operation, although it has some challenges associated with scaling up, particularly related to molecular weight and size considerations (Kim et al., 2016). This easy-to-use technique with relatively short separation times is often used to separate EVs from relatively dilute biological samples (Zhang et al., 2021). However, it has not addressed the problems of protein contamination and EVs membrane loss. There are also methods such as tangential flow filtration (TFF) and flow field-flow fractionation (FFF), all of which have limitations, such as the limited volume of samples to be processed. Kim et al. developed a novel dual-cyclic tangential flow filtration (dcTFF) chip to enable the efficient and selective isolation of highly purified, concentrated EVs (Figure 2C) (Kim et al., 2021). The proposed dcTFF system utilizes two membranes connected to a peristaltic pump to facilitate continuous circulation of the sample stream flowing to the membrane. The dual cyclic filtration approach used in the dcTFF system can isolate the specific EVs size range of 30–200 nm in one step, which is superior to direct filtration (DF) and single cyclic TFF (scTFF) methods. FFF is a versatile method for isolating EVs that offers a wide variety of eluent options and broad separation capabilities, but it requires specialized fractionation equipment and long duration due to considerations related to molecular weight and size (Sitar et al., 2015).

### 2.2 Charge-based techniques

**FIGURE 3:**
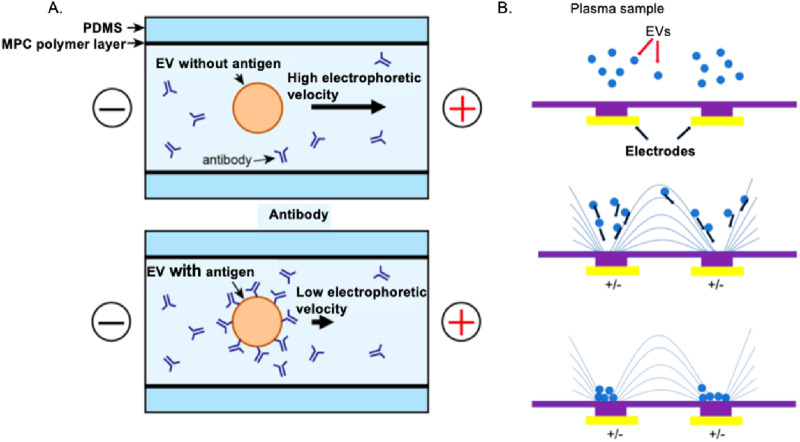
Electrophoresis and DEP to the charge-based separation of EVs. **(A)** Breast cancer cell-derived EVs can be effectively purified using on-chip microfluidic electrophoresis platforms (Akagi et al., 2015). **(B)** A dielectrophoretic-based microfluidic platform enables efficient isolation of EVs from A549 human non-small cell lung cancer cells (Chen et al., 2019).

Ion exchange describes a specific chemical process in which EVs in a biofluid to be tested, like a cell culture medium, is exchanged with other ions having a similar charge. Its application is limited by its use in matrices containing complex charged biomolecules (Wang et al., 2023), so it is often used to optimize other separation techniques and further improve the purity of EVs. Another method is to use electrophoresis and dielectrophoresis (DEP) to separate EVs and their subgroups based on their electrophoretic mobility (Morani et al., 2020). For instance, a microfluidic-based on-chip immunoelectrophoresis technique demonstrated in Figure 3A can be applied for effective differential protein expression profiling of individual EVs (Akagi et al., 2015). This method involves electrophoretic experiments on EVs derived from MDA-MB-231 human breast cancer cell culture supernatant using a microcapillary electrophoresis chip and a laser dark-field microimaging system. In Figure 3B, Chen et al. have introduced a straightforward and user-friendly DEP technique for isolating EVs that offers higher recovery efficiency (>83%) and purity compared to the traditional method of ultracentrifugation (UC) (Davies et al., 2012). The DEP chip can realize isolation within 30 min, much faster than the 8 h duration required for the UC procedure. There are several different categories of electrophoresis, each with its advantages and limitations. Some of the most common types of electrophoresis are gel electrophoresis and capillary electrophoresis. This method can be implemented by imposing a microfluidic chip with a significantly high recovery rate (Davies et al., 2012).

### 2.3 Affinity-based techniques

On the surface of EVs, proteins, lipids, and polysaccharides are exposed as potential ligands available for selection by antibodies, lipid-binding proteins, and lectin for molecule-specific interactions with molecules that can isolate EVs. Immunoaffinity-based methods involve antibodies targeting specific EVs surface markers such as CD63, CD9, and CD81. In direct methods, EVs are captured directly by immobilized antibodies, while in indirect methods, EVs are first labeled with antibodies and then captured by immobilized secondary antibodies (Duijvesz et al., 2015). Immunoaffinity-based methods offer high specificity for EVs, and different subpopulations of EVs can be isolated by targeting different surface markers. However, these methods can be costly due to the high price of antibodies, and the isolation efficiency may alter depending on the quality of the antibody used. Immunoaffinity assays for EVs isolation can use submicron-sized immuno-MBs to improve specificity, sensitivity, and yield. This enhancement results from a larger surface area, no sample volume constraints, and a near-homogeneous capturing process (Liangsupree et al., 2021).

Another affinity method that is commonly used for EVs isolation is lectin-based capture. Lectins are carbohydrate-binding proteins that can bind to specific sugar moieties on the surface of EVs. This method involves using lectin-coated beads or columns to capture the EVs. Plant-derived lectins such as concanavalin A (ConA) and wheat germ agglutinin (WGA) have been used to isolate EVs. ConA is a lectin that binds to the -D-mannose and -D-glucose residues found on the surface of EVs (Krafft et al., 2017; Lennon et al., 2019). Conversely, WGA binds to the N-acetylglucosamine and sialic acid residues found on the surface of EVs. The advantage of lectin-based capture is that it can capture a broader range of exosomes, as lectins can bind to various sugar moieties on the EVs surface. However, the disadvantage of lectin-based capture is that it may also capture non-specific particles that express similar sugar moieties.

Chemical affinity-based methods involve using chemicals that interact with specific EVs components, such as phosphatidylserine and cholesterol. Phosphatidylserine is a phospholipid exposed on the outer membrane of many EVs, making it a common target for chemical affinity-based isolation methods. One example of a chemical that binds to phosphatidylserine is lactadherin, a protein with a high affinity for this phospholipid. Cholesterol is another component abundant in the membranes of many types of EVs. Cyclodextrins are cyclic sugars that have been shown to bind to cholesterol and can be used for EVs isolation. Lactadherin and cyclodextrin can be immobilized on a solid support to isolate EVs from biological samples (Hansen et al., 2020; Zidovetzki and Levitan, 2007). These methods are simple and effective, and many EVs can be isolated. However, the specificity can be limited, leading to the isolation of non-specific contaminants.

### 2.4 Commercial kits and platforms for EVs isolation

Besides the conventional isolation methods, there are also commercial kits available for EVs isolation, catering to different research needs and sample types (e.g., plasma, serum, cell culture media). These kits vary in their underlying isolation principles, including size exclusion, ultracentrifugation alternatives, precipitation, and immunoaffinity. Table 2 lists some commonly used kits for EVs isolation.

**TABLE 2:** Commercial used kits and platforms of EVs isolation.

Kit name	Company	Principle	Pros	Cons	References
ExoQuick™	System biosciences	Polymer-based precipitation	Fast, user-friendly	Risk of isolating contaminants	Lobb et al. (2015)
Total exosome isolation	Thermo fisher scientific	Precipitation reagent	Straightforward protocol	Risk of non-specific co-precipitation	Takov et al. (2019)
qEV columns	IZON science	Size-exclusion chromatography	High purity, reproducibility	Requires concentration steps	Vogel et al. (2016)
Exosome isolation kit	Miltenyi biotec	Immunoaffinity (MBs)	High specificity	Limited yield, biases for specific EVs	Hashemi et al. (2024)
ExoEasy™ maxi kit	QIAGEN	Membrane affinity spin columns	High yield, good purity	High cost, specialized buffer required	Witwer et al. (2013)
Exo- spin™	Cell guidance system	SEC + precipitation	Balances purity and yield	Requires concentration steps	Enderle et al. (2015)

## 3 MBs-based EVs isolation

Since the traditional EVs isolation methods suffer from drawbacks like low accuracy, low efficiency, low specificity, high cost, and restricted applications, it is worth mentioning that using MBs to isolate EVs is an attractive strategy due to MBs’ unique properties such as ease of use, high efficiency, and reproducibility. The working mechanism of using MBs to isolate EVs is to attach a specific biomarker that could recognize and capture EVs onto the surface of MBs, and after coupling the EVs with the biomarkers on the surface of MBs, magnetic separation would isolate the EV-MB compounds from the background components. In this part, the basic concept of MBs, the synthesis and surface modification methods are firstly introduced. Then EVs isolation methods based on MBs are discussed. Finally, the strategies to improve the EVs isolation efficiency are proposed.

### 3.1 Basic concept of MBs-based EVs isolation

MBs are small magnetic particles composed of a core usually made of a metal compound and functional surface molecules. MBs have been applied in many biomedical engineering applications such as drug delivery, real-time monitoring, and cell separation due to their properties like high surface-to-volume ratio, high specificity, high sensitivity, and so on. MBs can be divided into different classifications according to different criteria. For example, based on the surface functionalization, the MBs can be classified into antibody-coated beads, streptavidin-coated beads, and aptamer-coated beads. Based on the size, the MBs can be divided into micro-sized beads and nano-sized beads. According to the main composition, the MBs can be classified into metal-based cores and polymer-based cores. In the following part, the synthesis methods, surface modification strategies are introduced as the basic preparation work before using MBs to separate EVs.

### 3.2 MB synthesis strategies

Based on synthesis process mechanisms, the synthesis method can be divided into three categories: physical methods, chemical methods, and biological methods. From the view of the change in size, MBs synthesis methods can be classified into top-down methods and bottom-up methods. Commonly, the physical methods are top-down methods since the synthesis process is to reduce the precursor material size through mechanical action, and biological and chemical synthesis are included in bottom-up method since these synthesis methods are to gather the smaller particles together to achieve the desired size (Nicule et al., 2022). Table 3 summarizes some common synthesis methods for MB.

**TABLE 3:** Common synthesis methods for MBs-.

Synthesis methods	Purity	Product beads size	Advantages	Disadvantages	Synthesis methods	Purity	Product beads size	Advantages
Ball milling	Moderate	100 nm–10 µm	Simple, cost-effective, Scalable	Broad size distribution, contamination	Co-precipitation	Moderate	5 nm–100 nm	Simple, cost-effective, scalable
Electron beam deposition	High	10 nm–200 nm	Excellent control over size and morphology	Limited to small-scale production, Expensive equipment	Hydrothermal	High	10 nm–1 µm	High crystallinity, Uniform size
Laser ablation	High	10 nm–1 µm	High purity, good control over size	High energy consumption, expensive equipment	Microemulsion	High	5 nm–50 nm	Good control over product size
Ion beam sputtering	High	10 nm–100 nm	Precise size control, highly pure	Expensive equipment, limited scalability	Sol-gel	High	10 nm–500 nm	High purity, tunable surface chemistry
Sonochemical method	High	5 nm–100 nm	Rapid synthesis, uniform size	Requires ultrasound equipment	Microwave-assisted synthesis	High	5nm–100 nm	Rapid synthesis, uniform particle size
Biological synthesis	Moderate	10 nm–500 nm	Eco-friendly, biocompatible	Low yield, limited scalability	Electrochemical synthesis	High	10 nm–1 µm	Precise control over size and composition, scalable

#### 3.2.1 Physical methods

Various physical methods are employed for nanoparticle synthesis, each with distinct mechanisms and applications. Ball milling is a mechanical technique that reduces particle size by grinding precursors into nanosized particles, often with added solvents or salt species to prevent agglomeration (Nicule et al., 2022; Balasubramanian et al., 2022). Electron beam deposition, also known as electron beam lithography, involves converting metal materials into oxides under exposure to an electron beam; nanoparticles are formed by evaporating the target material onto a resist-coated substrate (Reddy et al., 2012; Fu et al., 2018; Wnuk et al., 2011; Huth et al., 2018). Laser ablation utilizes a high-intensity laser beam to remove material from a solid surface, leading to the formation of nanoparticles or nanostructures (Piotto et al., 2023; Kim et al., 2017). Ion beam sputtering or ion beam deposition, uses energetic ion beams to sputter material from a target, resulting in the synthesis of magnetic nanoparticles under controlled conditions (Han et al., 2024).

#### 3.2.2 Biological methods

For the biological synthesis of magnetic nanoparticles, there are two main strategies: microorganism-based synthesis and plant-based synthesis. For microorganism-based synthesis, magnetostatic and iron-reducing bacteria can produce, either intracellularly or extracellularly, single-domain MNPs under anaerobic conditions (Marcelo et al., 2020). Fungi release enzymes and proteins that act as reducing agents, facilitating the synthesis of metal nanoparticles from metal salts (Jamkhande et al., 2019). In plant-based synthesis, the bioactive molecules found in green materials can serve dual roles as reducing and stabilizing agents, ensuring the stability of nanoparticles during formation. This approach enables precise control over nanoparticle size and shape, opening up diverse application possibilities (Yew et al., 2020).

#### 3.2.3 Chemical methods

Several chemical methods are employed for synthesizing magnetic nanoparticles (MNPs), each offering distinct advantages. The co-precipitation method is one of the simplest and most widely used, where Fe^2+^ and Fe^3+^ salts react in an alkaline solution to form Fe_3_O_4_ nanoparticles under oxygen-free conditions (Marcelo et al., 2020; Dalili et al., 2018; Liu et al., 2020). The sonochemical process, also known as ultrasonic or acoustic cavitation, is a variation of co-precipitation that utilizes ultrasound to produce cavitation bubbles, which in turn generate nanoparticles (Marcelo et al., 2020; Qi et al., 2022; Aliramaji et al., 2015). The hydrothermal method involves the reaction of aqueous solution vapors with solids under high pressure and temperature, resulting in enhanced crystalline structures such as magnetite nanorods and nanospheres (Jamkhande et al., 2019; Liu et al., 2020). Sol-gel synthesis begins with the hydrolysis of precursors to form a colloidal sol, which transitions into a gel and is further treated to obtain nanomaterials (Nicule et al., 2022; Marcelo et al., 2020; Navas et al., 2021). The microemulsion method involves dissolving MNP precursors in the oil phase of a microemulsion, stabilized by surfactants, to create a homogeneous environment for forming uniform-sized nanoparticles (Carinelli et al., 2023). Microwave-assisted synthesis uses microwave radiation to accelerate chemical reactions, enabling rapid and uniform formation of nanoparticles with reduced processing times (Gupta et al., 2018). Lastly, electrochemical synthesis is based on redox reactions involving an iron-based sacrificial electrode, allowing precise control over nanoparticle size and crystallinity through the application of electric current (Marcelo et al., 2020; Cao et al., 2024; Chaplin, 2018).

### 3.3 Surface modification of MBs for EVs isolation

Surface modification is a crucial process that enhances the functionality of MBs by altering their surface properties to improve anti-fouling, stability, specificity, and performance in various applications. It involves the introduction of chemical or biological moieties onto the bead surface, enabling selective interactions with target molecules such as EVs. There are several approaches to surface modification, broadly classified into physical adsorption, covalent bonding, and bioaffinity interactions. Physical adsorption relies on electrostatic or hydrophobic interactions to attach biomolecules, but may suffer from weak stability. Covalent bonding creates strong and durable linkages between the bead surface and functional molecules, such as antibodies or polymers, ensuring long-term stability. Bioaffinity interactions, such as biotin-streptavidin or protein A/G conjugation, provide highly specific and reversible binding, making them useful for controlled capture and release applications.

#### 3.3.1 Antifouling

In the isolation of EVs, sample impurities and fouling pose significant challenges. This is because in real biological samples, nonspecific adsorption of impurities like proteins can lead to false positives or false negatives, which is a critical issue in many sensor analysis systems. As a result, anti-fouling has emerged as a crucial component of sensor analysis strategies. To improve the efficiency of EVs isolation, it is essential to prevent nonspecific binding. In Table 4, we list some antifouling strategies for MBs.

**TABLE 4:** Antifouling strategies of magnetic nanoparticles.

Antifouling strategies	Nanostructure	Antifouling moiety	Application	Antifouling strategies	Nanostructure	Antifouling moiety	Application	References
PEGylating strategies	Metal-phenolic NPs (MPs)	PEG	SPECT/PET	Zwitterionic strategies	poly (cyclotriphosphazene-copolyethylenimine) nanospheres (PNSs)	1,3-PS	Theranostic	Zhu et al. (2019), Li et al. (2015)
	Magnetic iron oxide NPs (IONPs)	PEG and allyl glycidyl ether (PEG-b-AGE)	Theragnostic					Wang et al. (2019a), Suarez-Garcia et al. (2021)
	Hybrid lutetium oxide NPs (UCNPs)	PEG	Multimodal imaging [up-conversion luminescent, X-ray and Magnetic Resonance Imaging (MRI)]		Janus dendrimer (JD GPC) Au DENPs	Glycerylphosphorylch oline (GPC)	Targeted drug	Li et al. (2020), Liu et al. (2014)
	Poly (oxyethylene galactaramide)s (PEGA) NPs	PEGA	OI		Au NPs	Peptide sequence of glutamic acid and pCBMA	Radiotherapy	Ding et al. (2020), Jeong et al. (2013)
	poly (ethyleneimine)	PEG	Drug delivery		poly (2-(diisopropylamino)ethylmethacrylate) (PDPA) NPs			Ding et al. (2019), Park et al. (2015)
Zwitterionic strategies	IONPS		MRI		Dendrimer-entrapped gold (Au DENPs)	Carboxybetaine acrylamide (CBAA)	Gene delivery Photothermal drug delivery	Xiong et al. (2019), Ferretti et al. (2021)
	Mn_3_O_4_ NPs	L-lysine	MRI		Poly (N-isopropylacrylamide) (PNIPAM) Nanogels	Sulfobetaine methacrylate (SBMA)		Zheng et al. (2020), Wang et al. (2019b)
	Ultrasmall Gadolinium oxide (NPs Gd2O3 NPs)	PEG-L-cysteine	MRI	Other strategies	IONPs	Dendron coating	Theragnostic	Cotin et al. (2019), Sui et al. (2020)
					Silver NPs (Ag NPs)	Chitosan and dextran	Targeted drug therapy	Shi et al. (2018), Sui et al. (2020)
					Etherified starch-coated	Amphoteric starch	Targeted drug	Sui et al. (2020), Chrastina et al. (2011)
					Multifunctional peptides	Antifouling domain	Biosensing	Liu et al. (2018a), Sui et al. (2020)

**FIGURE 4:**
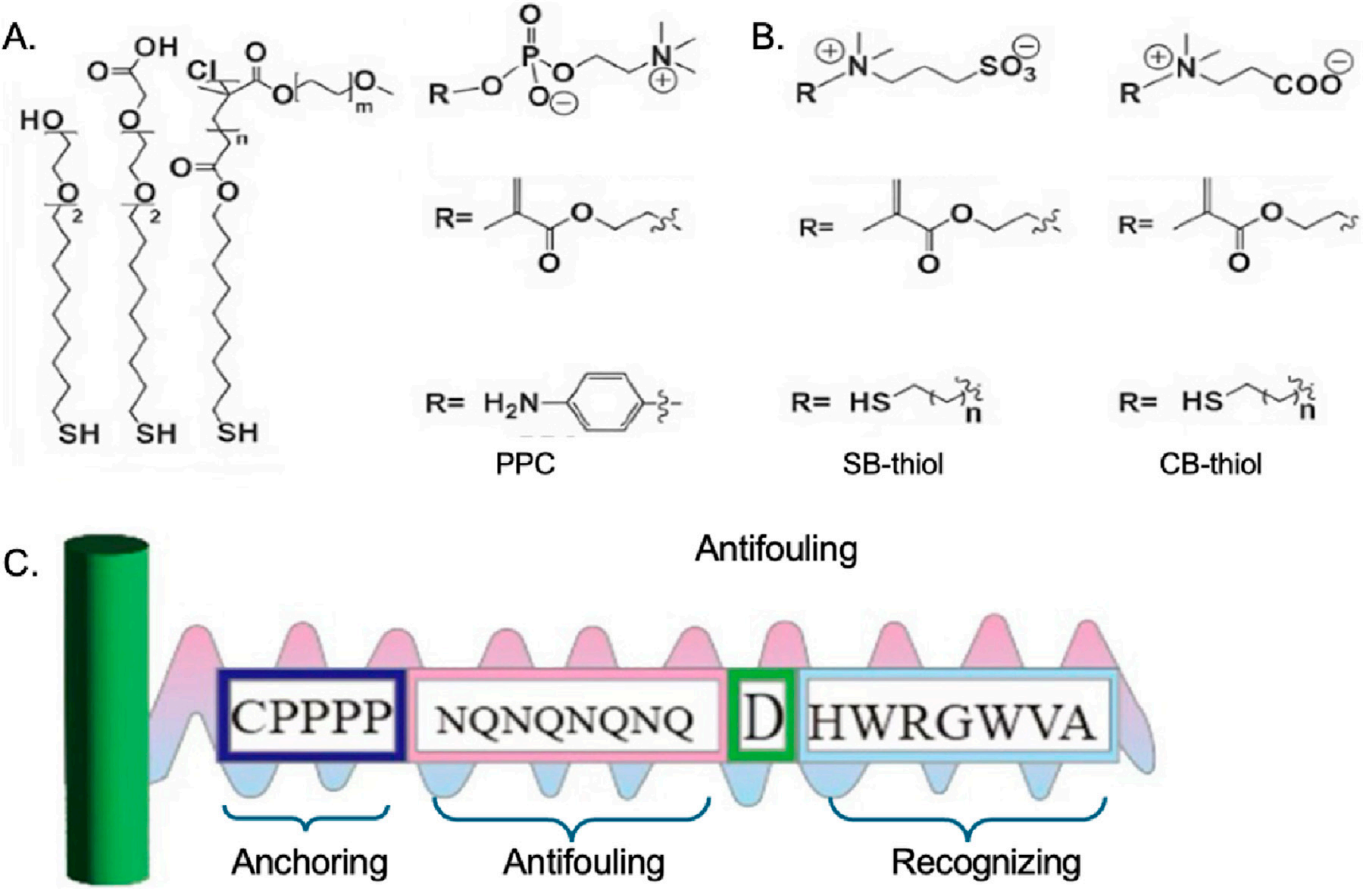
Chemical structures of **(A)** OEG/PEG based thiolds (Jiang et al., 2020), **(B)** zwitterionic molecular system (Jiang et al., 2020), **(C)** multifunctional peptide (Liu N. et al., 2018).

One approach is to modify the MB surface with polymer coatings or zwitterion molecules that prevent the nonspecific binding of molecules and enhance the hydrophilicity of the beads (Figure 4). Polyethylene glycol (PEG) is a common polymer used for this purpose. PEGylation of the MBs can reduce nonspecific protein adsorption, enhance biocompatibility, and increase the stability and colloidal stability of the beads in complex biological fluids (Br et al., 2022). Other polymers such as poly (ethyleneimine), poly (lactic acid), and poly (glycerol methacrylate) have also been reported to improve the performance of MB-based EVs isolation (Multia et al., 2019; Li et al., 2018). Since the 1970s, polyethylene glycol (PEG) and its derivatives are undoubtedly the most easily available and most frequently used antifouling materials, and they have been used in various forms of sensors PEG-based SAM sensors are very common (Ostuni et al., 2001). For example, the recently developed multi-channel SPR sensor is used to simultaneously detect three human pancreatic peptide hormones, namely, insulin, glucagon, and somatostatin (Castiello and Tabrizian, 2018). The antifouling mechanism of PEG is not yet fully understood, but it is primarily attributed to three factors: (1) its low interfacial energy, (2) hydration resulting from hydrogen bonding, and (3) the mobility and flexibility of PEG chains. The PEG has a unique and effective ability to resist protein adsorption (or blood compatibility). Among the anti-pollution materials currently used, PEG is the most widely used and has the best anti-pollution effect. Therefore, PEG is often used as a “ruler” to measure the anti-pollution performance of other materials (Xing et al., 2017). However, PEG has some drawbacks like the susceptibility to oxidative damage, easy decomposition in the presence of oxygen and transition metal ions such as Zn^2+^, and loss of its anti-protein adsorption performance, which affects the anti-fouling performance and the stability of MBs for long-term application.

Zwitterionic material refers to polymer molecules that contain both positive and negative ions, which possess high oxidative resistance and hydrolytic stability, have attracted considerable attention as promising alternatives to PEG in developing high-performance antifouling inter-faces (Lowe and McCormick, 2002). Zwitterionic groups can be in small molecules, such as lecithin; it can also be in polymers such as polybetaines. Among them, polybetaine is an important class of zwitterionic polymers. Zwitterions contain the same number of positive and negative charges, so they are neutral. And because of the super-hydrophilic properties of zwitterions, it can effectively resist nonspecific adsorption or “contamination” from biomolecules and microorganisms, thereby preventing foreign body reactions (He et al., 2016). Although zwitterionic compounds show excellent anti-fouling abilities, the availability of these kinds of materials is limited since few zwitterionic compounds are commercially available, while the synthesis and purification process are complicated. Besides, the zwitterionic compounds are susceptible to the external PH and electric field, which may also have a negative influence on the anti-fouling behavior.

Another material used as an anti-fouling molecule is peptides (Liu N. et al., 2018). Peptides typically exhibit strong hydration due to the high hydrogen bond-donating and -accepting capabilities of their polar functional groups, as well as their zwitterionic nature. The peptide-based anti-fouling interfaces in sensors can be rationally designed for a given specific applications the chemical tunability (i.e., amino acid sequence) of the peptide allows it to be applied into various kinds of interfaces to perform the anti-fouling ability. What is more, compared to PEG and zwitterionic materials, peptides show better *in vitro* stability which is in favor of long-term application.

Silver nanoparticles (AgNPs) are the most effective antibacterial agents because they are highly resistant to viruses, microorganisms, and other eukaryotic microorganisms. However, a smaller specific surface area tends to reduce the binding capacity of AgNPs to bacteria, AgNPs can destroy and kill bacteria by attaching to the cell membrane (Kim et al., 2018). So, AgNPs can be added to the antifouling material to improve its bactericidal performance. Some researchers have developed a new anti-fouling and anti-infection hydrogel, providing a promising strategy for promoting chronic wound healing (Shi et al., 2018).

#### 3.3.2 Stabilization

Stabilization of MBs is a critical aspect of their design, ensuring their long-term performance, colloidal stability, and reliability in various biological and analytical applications. MBs tend to aggregate due to van der Waals forces and magnetic dipole interactions, which can compromise their dispersion, reactivity, and binding efficiency. To prevent this, stabilization strategies involve surface modifications that enhance repulsive forces and reduce bead aggregation.

Organic coating, for example, polymers like PEI, PAA, polydopamine, polyphenol, and polyelectrolytes have many functional groups that have the electrostatic repulsion effect and could stabilize the particles by helping the particles disperse evenly in the solvent. Membrane coatings like myeloid-derived suppressor cell membrane coating and magnetosomes (membrane-enveloped MBs, biomineralized by magnetostatic bacteria) could equip the MBs with the biological characteristics, including the surface receptor and antibodies that can possibly be used for immune evasion and active targeting of tumor cells or the tumor microenvironment, while increasing the stability of isolated particles.

Inorganic coatings, such as metals, could be coated on the magnetic nanoparticles. These coating materials, like gold and platinum, could make the MNP with super magneticity and ease of detection in the human body. What’s more, the coating could prevent the core from oxidation, and it can enable the grafting of biomolecules. Silicon dioxide coating has general advantages like increase the biocompatibility. Besides, it could provide cross-linking bonds for the MNP, and it can form an inert external shielding layer to protect the NPs.

### 3.4 Influence of synthesis and modification methods on EVs isolation outcomes

The performance of MBs in EV isolation is fundamentally dictated by their synthesis route and surface functionalization, which collectively influence capture efficiency, purity, and specificity. In this chapter, we discuss how synthesis and modification methods influence MB-based EVs isolation. Furthermore, we compare the effects of these various synthesis and modification methods on EVs isolation efficiency in Table 5 for clearer visualization (Jiang et al., 2020).

**TABLE 5:** Different MBs synthesis methods’ suitability for EVs isolation.

Synthesis method	Synthesis method classification	EVs isolation efficiency	Repeatability	Suitability for EVs isolation
Ball milling	Physical method	Extremely low	Extremely low	Extremely poor
Laser ablation	Physical method	Low	Moderate	Poor
Electron beam deposition	Physical method			Not applicable
Co-precipitation	Chemical method	Moderate	Moderate	Good
Hydrothermal synthesis	Chemical method	High	High	High
Magnetosomes	Biological method	Moderate	Extremely high	Poor

#### 3.4.1 Influence of MBs synthesis methods for EVs isolation

Firstly, different synthesis methods will affect the EVs isolation efficiency. MBs synthesized *via* physical methods are more like raw materials for subsequent biomedical applications rather than precision-engineered devices. The characteristics of MBs synthesized by physical methods are not compatible with the requirements for high-efficiency and high-purity EV isolation.

Physically synthesized MBs are unsuitable for EVs isolation because of their uncontrolled surface chemistry, irregular size distribution, unstable magnetic properties, poor dispersion stability and limited functionalization potential. The physical synthesis methods (e.g., ball milling) inevitably introduce impurity contamination during fabrication, resulting inMBs with complex and inert surface chemistry. This defective surface state induces strong nonspecific binding, ultimately yielding EVs with compromised purity that are unsuitable for downstream analytical applications. Moreover, the irregular morphology and polydisperse size distribution characteristic of physically synthesized MBs lead to significant variability in EVs capture capacity. This inherent heterogeneity results in both inconsistent capture efficiency and non-quantifiable binding performance, fundamentally compromising the reproducibility of EV isolation protocols. Additionally, physical methods like ball milling severely damage the crystal structure during synthesis, weakening the magnetic properties. This forces reliance on extremely high magnetic field strengths for separation, resulting in low efficiency. Besides, the absence of a stable coating on the surface of physically synthesized MBs makes them prone to aggregation, reducing the effective surface area and consequently lowering the capture efficiency. Finally, physically synthesized MBs lack reactive functional groups on their surfaces, and contaminating impurities further hinder subsequent chemical modifications, resulting in significant challenges for EV separation.

The biologically synthesized MBs (magnetosomes) theoretically represent the most ideal synthetic approach for EV separation, exhibiting exceptional biocompatibility and purity. Their natural phospholipid membrane, compositionally similar to cell membranes, ensures superior compatibility with biological systems. The extremely low nonspecific adsorption enables isolation of high-purity EV samples, which is critical for downstream analyses by significantly reducing background interference.

However, biologically synthesized MBs face significant practical limitations in real-world applications. This synthesis method suffers from extremely low yields and prohibitively high costs. Moreover, the extraction and purification processes for biosynthetic MBs are exceptionally complex. Additionally, functionalizing their phospholipid membranes proves more challenging compared to conventional carboxyl- or amino-modified MBs. Consequently, despite magnetosomes’ theoretically ideal properties for EV separation, they remain rarely employed in practical applications.

Therefore, chemical synthesis methods currently represent the optimal approach for EV separation. In chemical synthesis methods (e.g., coprecipitation, hydrothermal synthesis), MBs formation occurs through a concurrent process. The magnetic core formation and *in situ* encapsulation by surfactants or polymers proceed simultaneously, yielding complete and stable final products. By precisely tuning the reaction parameters, both the size and surface chemistry of the MBs can be controllably engineered. Moreover, the bead surfaces can be further modified through silanization or polymer grafting methods to introduce specific functional groups for further applications.

To conclude, MBs produced by physical synthesis methods are primarily designed for macroscopic separation applications (e.g., wastewater treatment), where possessing magnetic properties alone suffices. In contrast, EV separation requires beads that not only exhibit magnetic responsiveness but also require biocompatibility, surface purity, precise functionality, size uniformity, excellent stability. Although MBs synthesized *via* biological methods show perfect potential, the practical limitations make them not suitable for EVs isolation. Therefore, chemical synthesis methods derived MBs are the best choice for high-efficiency and high-purity EVs isolation.

#### 3.4.2 Influence of surface modification method for EVs isolation

In addition to the synthesis method, subsequent surface modifications also significantly impact the efficiency of EV isolation. For example, silica-coated MBs provide surface Si-OH groups, which serve as a foundation for subsequent silanization to introduce high-density functional groups, thereby indirectly enhancing EV isolation efficiency. PEG- and zwitterion-modified MBs can effectively block nonspecific adsorption by forming a hydration layer, thereby significantly improving separation purity. Silanization and polymer grafting provide abundant functional group binding sites, significantly enhancing EV separation efficiency.

In summary, surface modification reduces nonspecific adsorption, thereby improving EVs. Isolation efficiency. Concurrently, surface modifications provide abundant binding sites on the beads, introducing functional groups (e.g., carboxyl, amine) and streptavidin, which increase antibody conjugation capacity and directly enhance EV separation performance. A growing body of evidence indicates that both the synthetic design of MBs and their surface functionalization strategies are pivotal determinants of EVs isolation efficiency. For instance, Hu and Gao showed that aptamer–nanomaterial functionalization integrated into MB-based microfluidic platforms substantially enhanced the yield of exosome capture, thereby demonstrating the critical role of surface modifications in improving performance (Hu and Gao, 2025). In line with this, Ma et al. synthesized Fe_3_O_4_@ZrO_2_ composite MBs that exploit ZrO_2_–phosphate interactions and reported significantly improved recovery rates and sample purity, highlighting how material composition and fabrication pathways directly influence separation outcomes (Ma et al., 2024). Supporting this observation, Grishaev et al. found that tannic acid–coated CaCO_3_/Fe_3_O_4_ beads enabled efficient recovery of small EVs while maintaining vesicle integrity, emphasizing the importance of coating chemistry for balancing efficiency with structural preservation (Grishaev et al., 2024). Extending beyond single-experiment findings, Solovicová et al. systematically reviewed recent progress in magnetic affinity-based separation and concluded that different ligands (such as antibodies, aptamers, peptides, glycans, and phospholipid-binding moieties) lead to pronounced differences in capture efficiency, specificity, and reproducibility (Solovicová et al., 2025). Consistently, Cui et al. (2024) developed aptamer-functionalized Fe_3_O_4_@Ti_3_C_2_ MXene MBs and demonstrated that coupling advanced composite materials with aptamer modification markedly improves both enrichment and downstream detection of EVs (Cui et al., 2024). Taken together, these studies strongly support the argument that variations in bead synthesis, coating chemistry, and ligand choice exert decisive effects on EV isolation efficiency, and should therefore be carefully considered when designing separation platforms for diagnostic or therapeutic applications.

## 4 MBs-assisted platforms for EVs isolation

### 4.1 Sole MBs

MBs are often chosen as the solid media when conjugate ligands like antibody to develop the affinity-based isolation technique. Take the antibody-based affinity method as an example. Briefly, the procedure of this isolation method is that the antibody-conjugated MBs are first incubated with the biological fluid. After the antibody captures the EVs, discard the supernatant and collect the EV-MB conjugates. The next step is to resuspend the EV-MB conjugates and incubation for EVs elution. Finally, the MBs are removed by magnetic separation, and the EVs are collected in the supernatant (Chen et al., 2020).

Xu’s research team introduced a MB-based method for EVs isolation, which achieves twice the yield and comparable purity of harvested EVs compared to ultracentrifugation (UC) (Fang et al., 2021). This method is called MB-mediated selective adsorption strategy. The brief procedure is shown below. First, incubate the mixture of MBs, stock solution, PBS, and biological fluid. After the incubation, discard the supernatant and disperse the MBs-EV conjugates for EVs elution. The last step is to remove the MBs by magnetic separation, and the harvested EVs remain in the supernatant. Although this isolation method generates a much higher yield than the UC method, there are still improvements that could be optimized to improve the yield.

### 4.2 MBs in microfluidics

**FIGURE 5:**
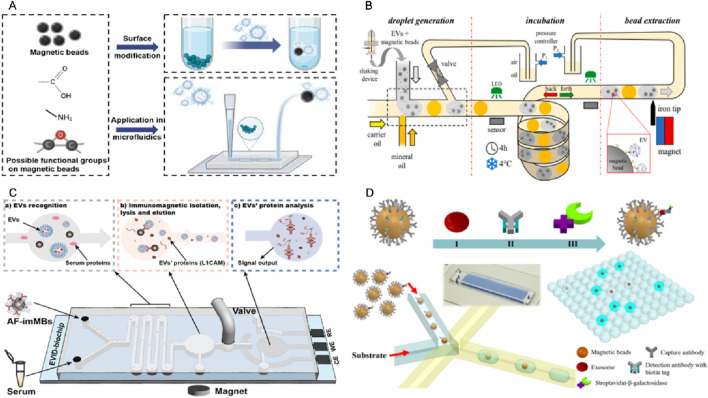
Platforms for magnetic bead-based EVs isolation in microfluidics. **(A)** Schematic drawing showing the functionalization of MB for EV isolation and its application in microfluidics. **(B)** A microfluidic chip consisting of the droplet generator, incubator, and magnetic bead extraction module for EVs isolation (Meggiolaro et al., 2024). **(C)** An integrated microfluidic chip acts as a generic liquid biopsy platform to isolate EVs and detect transmembrane protein L1CAM of EVs (CD81-positive) (Li et al., 2024). **(D)** A microfluidic chip for digital quantification exosomes which was captured by antibody modified MBs (Liu C. et al., 2018).

Compared to solo MBs EVs isolation, the MB-based microfluidic isolation significantly improves the EVs recovery rate, reduces the reagent volume, and cuts the operation time. The basic principle is shown in Figure 5A. Microfluidic designs create controlled laminar flow and tunable shear forces, which enhance the probability of contact between EVs and functionalized MBs by minimizing turbulence and promoting uniform trajectories. Within confined microchannels, the spatial distribution of beads can be precisely optimized, thereby reducing aggregation and ensuring a higher effective surface area for vesicle capture. When coupled with external magnetic fields, the microfluidic environment enables dynamic manipulation of bead positioning and movement, allowing selective retention of target EVs while unbound impurities are washed away efficiently. This synergy between microfluidics and MBs not only improves capture efficiency and specificity but also enhances reproducibility and scalability by enabling continuous, automated, and high-throughput separation. Collectively, these mechanistic insights highlight how the convergence of fluid dynamics and magnetic control provides a powerful framework for advancing EV isolation technologies.

Ferraro’s team developed an automated droplet microfluidic platform (Figure 5B) that integrates on-chip droplet incubation with in-line magnetic extraction to enable efficient and reproducible immunomagnetic isolation of EVs from large-volume samples (up to 2 mL). The captured EVs were validated by protein assays, nanoparticle tracking, and miRNA analysis. This strategy demonstrated shorter incubation times and substantially higher capture efficiency than conventional batch protocols (Meggiolaro et al., 2024). We recently developed an integrated biochip platform (Figure 5C), which integrates *in situ* electrochemical protein detection with on-chip antifouling-immunoMBs modified with CD81 antibodies and zwitterion molecules, enabling efficient isolation and detection of neuronal EVs. The capability of the system to isolate common EVs and detect neuronal EVs associated with Parkinson’s disease in human serum is successfully demonstrated, using the transmembrane protein L1-cell adhesion molecule (L1CAM) as a target biomarker. For the first time, this study discovered that the level of L1CAM/neuronal EVs particles in serum could serve as a reliable indicator to distinguish Parkinson’s disease from control groups with AUC = 0.973. This approach has the potential to advance the diagnosis and biomarker discovery of various diseases (Li et al., 2024). Besides EVs isolation, microfluidic chip can also help with EVs signal output. Zheng’s team implemented a droplet digital ExoELISA (Figure 5D) in which individual exosomes were captured on antibody-functionalized MBs and labeled with an enzyme-tagged detection antibody. The capture exosomes on MBs were co-encapsulated with substrate into microdroplets to produce fluorescent readouts that were enumerated and converted *via* Poisson statistics to yield absolute concentrations of protein-specific exosomes (limit of detection ≈5 exosomes μL^−1^). The clinical feasibility demonstrated by quantifying GPC-1–positive exosomes in breast cancer patient samples (Liu C. et al., 2018).

Mai’s team has refined a MB-based method that does not rely on antibodies. They initially optimized it in batch processing and later adapted it for use in microfluidic droplet systems (Morani et al., 2022). They greatly enhanced the reproducibility of EVs recovery and eliminated positive false bias by incorporating a washing step and optimizing the protocol. This approach significantly increases the recovery rate (up to 50%) while reducing sample and reagent volumes (by more than 10 times) and operation time (by three times) compared to the traditional batch mode.

**FIGURE 6:**
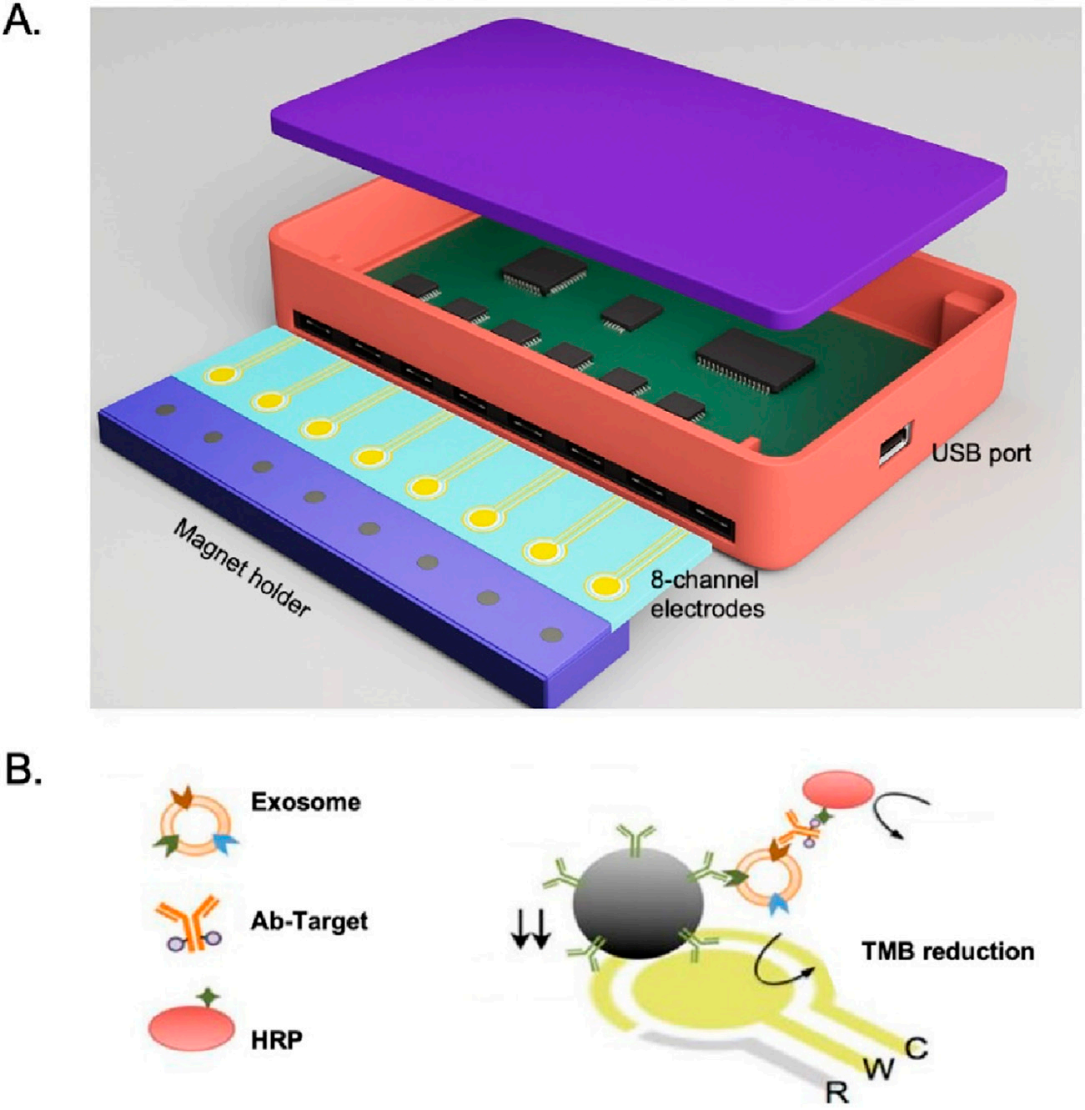
**(A)** Integrated magnetic–electrochemical exosome (iMEX) platform. The sensor is designed to measure signals from eight electrodes simultaneously. Small cylindrical magnets are positioned beneath the electrodes to concentrate exosomes captured through immunomagnetic methods. **(B)** Schematic of iMEX assay. Exosomes are captured directly in plasma using MBs, which are coated with antibodies specific to CD63. The captured exosomes are then labeled with horseradish peroxidase (HRP) for electrochemical detection. The system allows for simultaneous monitoring of eight channels, enabling high-throughput analysis (Jeong et al., 2016).

Several studies have demonstrated the successful application of integrated microfluidic chips to isolate and detect EVs in various diseases. One example is the successful implementation of an integrated microfluidic chip to isolate and detect prostate-specific membrane antigen (PSMA)-positive EVs in urine samples from prostate cancer patients (Zhao et al., 2016). The chip utilized a combination of PSMA antibody functionalized MBs for EVs capture, followed by RNA extraction and reverse transcription for PSMA mRNA detection, showing higher sensitivity than traditional ELISA methods in prostate cancer monitoring. Another study by Jeong et al. in Figure 6 shows that the chip integrated multiple functions into a single device, including EVs capture, washing, elution, and on-chip detection (Jeong et al., 2016). The chip consists of a microchannel coated with anti-CD63 antibody, which precisely captures EVs expressing CD63 on their surface. The captured EVs can be subsequently washed and eluted from the chip using a buffer solution. The eluted EVs can be directly analyzed on the chip using surface plasmon resonance (SPR) or fluorescence imaging. In addition to their diagnostic applications, integrated microfluidic chips have also been used in research on the role of EVs in diseases such as non-small cell lung cancer (NSCLC). Chen et al. developed a microfluidic chip for isolating and profiling EVs in NSCLC patients (Chen et al., 2023). The chip utilized a combination of immunoMB-based solation and surface-enhanced catalytic hairpin assembly imaging for EVs profiling. They identified a unique EVs signature in NSCLC patients that could serve as a biomarker for early-stage diagnosis.

### 4.3 Magnetic flowcytometry

Magnetic flow cytometry is an advanced method that leverages magnetic particles and sensors integrated into microfluidic structures for the dynamic analysis of fluid samples, which could be utilized to isolate EVs. It combines flow cytometry principles with magnetic separation to isolate specific cells like EVs. MBs are conjugated to antibodies targeting specific cell surface markers, so when these cells pass through a magnetic field, they are deflected and can be separated from the rest of the sample due to the interaction between the magnetic particles and the magnetic field, allowing for the separation, counting, and characterization without the need for optical detection methods (Bha et al., 2018).

## 5 Strategies to enhance separation efficiency for MB platforms

### 5.1 Experimental factors influencing efficiency

Specific to the research mentioned above, Xu’s group discovered that in their experiment, when polystyrene-based MBs are used as adsorption carriers for isolating EVs, the yield and purity are significantly lower compared to using silica-based MBs. This discrepancy might be attributed to the stronger adsorption properties of the polystyrene surface on proteins and EVs, driven by hydrophobic interactions. Additionally, EVs adsorbed onto polystyrene-based MBs are more difficult to elute compared to those on silica-based MBs. Also, through a controlled experiment, they found that with the same functional group (hydroxyl group), MBs with a diameter of 1 μm have a higher purity, and with the same diameter of 1 μm, the types of functional group on the MBs’ surface have little effect on the isolation purity. However, increasing the number of beads to increase the binding surface area could increase the isolation efficiency (Fang et al., 2021).

What is more, besides the optimization within one isolation strategy mentioned above, different isolation platforms also differ from others in efficiency. For instance, microfluidic isolation has advantages like lower volume, shorter incubation time, higher EVs recovery rate and purity over the conventional sole MBs in-batch isolation. For microfluidic isolation, Mai’s team conducted optimizations in several key aspects of their device. They explored the effects of varying MB concentrations (ranging from 0.5 to 2 mg/mL), the chemical surface of the beads (with carboxylic or silane groups), the incubation temperature for EVs capture (spanning from 4 °C to 25 °C), and PEG concentrations (between 5% and 15% w/v). They found that the MBs capture performance for the carboxylic group achieved EVs yield of 61%, which was significantly higher than silane group MBs with a yield of less than 5%, while the reaction temperature from 4 °C to 25 °C did not affect the EVs yield a lot. For the concentration of MBs, a too-high concentration would cause poor recirculation of the device since the beads would cluster within the channel. A high concentration of PEG would increase the viscosity, which limits the MBs’ EVs capture ability. Finally, the highest EVs recovery rate of 25% ± 8% was achieved with a PEG concentration of 5% and a carboxylic MBs concentration of 1.5 mg/mL with 1 h incubation at 25 °C. Besides, the moving pattern of droplets also affects the recirculation of beads in the microfluidic device. They proposed that the U-shaped move pattern could achieve the EVs recovery rate of 39% ± 3% (Morani et al., 2022). The development of integrated microfluidic chips for separating and detecting EVs has been an active area of research in recent years. Integrated microfluidic chips can combine multiple steps, such as EVs capture, washing, and detection, into one device. This allows for efficient isolation and analysis of EVs from biofluids with minimal sample and reagent consumption. In the meantime, the integrated microfluidic chip can provide an airtight space to avoid environmental contamination and achieve automatic operation.

### 5.2 Device-level optimization strategies

To improve the MB-based EVs isolation efficiency, there are several factors to take into consideration such as incubation time, temperature, the level of surface marker expression, and the concentration of target vesicles are important considerations. Additionally, the nature and state of the target molecule or structure, the characteristics of the antibody–antigen interaction, the type and concentration of the sample, and the ratio of beads to target molecules can all significantly impact the success of magnetic separation (Sioud, 2015). The detailed optimization depends on the specific reagents and methods used in the isolation process.

One strategy to improve EV isolation efficiency is to integrate isolation with downstream elution and analysis. Although there are commercial kits for MB-based EVs isolation like ExoCAS-2, which is easy to operate, there are no downstream lysis and analysis, which do not provide an elution process to recover the intact EVs, and the lack of universal EVs markers is another disadvantage for these strategies to ensure total capture of all EVs (Morani et al., 2022).

Another strategy to improve the EVs isolation efficiency is to build a multiplex bead-based platform that could achieve simultaneous capture of different EVs subpopulations. Stefan Wild’s group proposed a new multiplex bead-based platform has been introduced to analyze up to 39 different surface markers in a single sample (Koliha et al., 2016). This platform integrates capture antibody beads with fluorescently labeled detection antibodies. It enables the analysis of EVs that express surface markers recognized by both types of antibodies. Finally, they demonstrate that MBs can separate EVs mixtures and analyze them with the multiplex platform. The multiplex platform generates 39 different bead populations, each population of beads is conjugated with a distinct capture antibody, which specifically recognizes and binds to EVs that carry the corresponding antigen. With the present strategies to improve the EVs isolation efficiency, there are more advances could be achieved in the future. For example, the MBs used in the isolation can be further functionalized, introducing new ligands and using multi-marker strategies can significantly increase the isolation efficiency. Also, developing automation and high-throughput platforms like microfluidics and flow cytometry can improve efficiency by combining the isolation process with the downstream analysis.

In the future, more hybrid techniques platforms should be developed to utilize MBs with other conventional isolation methods to create new possibilities for improving the EVs isolation efficiency. Another important demand is to standardize the protocol for MBs-based EVs isolation techniques, achieving reproducible and repeatable protocols for different isolation platforms to be applied in clinical applications.

## 6 Advantages and challenges

MBs-based EVs isolation offers several advantages over conventional methods, such as size-based (e.g., ultracentrifugation) and charge-based (e.g., ion exchange) techniques. These advantages include higher specificity and efficiency through surface functionalization of beads with antibodies or ligands that selectively bind to EVs markers, reduced processing time, and the ability to work with small sample volumes. Additionally, magnetic isolation allows for easier automation, minimal equipment requirements, and improved reproducibility, making it a more convenient and scalable option for clinical and research applications. While immunoaffinity and aptamer-based capture offer high specificity and can isolate EVs subpopulations with specific marker proteins, this strategy still has several challenges that need to be addressed, such as non-specific binding, low yield, reproducibility issues, and elution difficulties.

### 6.1 Non-specific binding, low yield and sensitivity issues

One of the major challenges in EVs isolation using MBs is non-specific binding. Non-targeted biomolecules such as proteins, lipids, and other cellular debris would adhere to the beads ‘surface, leading to contamination, reducing the purity of the isolated EVs, and potentially interfering with downstream analyses. These non-specific bindings occur due to inadequate antifouling coatings or improper surface functionalization of the beads. Polymer-coated MBs, such as PEGylated or zwitterionic-coated beads, can overcome the challenge of nonspecific binding, but they may also alter the biological properties of EVs. For example, PEGylation can mask surface markers on EVs, making them less recognizable by target cells or tissues (Xing et al., 2017). Moreover, the high hydrophilicity of polymers could interfere with the binding of EVs to their natural targets *in vivo*, thus limiting their applications in drug delivery and therapeutics (Lowe and McCormick, 2002). While surface modifications with anti-fouling coatings can reduce these effects, achieving complete selectivity remains difficult. To address this, we propose several potential directions for resolution. Firstly, engineering surfaces with optimal spatial organization, controlling the density and chain length of the anti-fouling molecules to minimize steric interference, determine the optimal spatial arrangement and stoichiometric ratio between anti-fouling molecules and capture ligands. Secondly, developing stimuli-responsive coatings (e.g., pH-, temperature-sensitive) that can dynamically modulate the conformation of the surface molecules to facilitate capture and enhancing purification. For example, MBs are equipped with a dynamic coating of anti-fouling molecules in the buffer, and the coating will switch to a capture-active state when MBs are placed in sample media *via* a structural reconfiguration, which unmask the binding sites for high-efficiency EVs capture. In summary, MB-based EV isolation faces a trade-off between reducing nonspecific bindings and EVs isolation efficiency. Future efforts are needed to solve this situation by optimizing antifouling ligands spatial organization and developing stimuli-responsive coatings to dynamically balance capture efficiency with isolation purity.

### 6.2 Reproducibility and standardization

Reproducibility is a critical concern in EVs research. Even slight variations in isolation protocols can lead to inconsistent results. Factors such as differences in bead surface chemistry, incubation times, buffer compositions, and sample processing conditions can affect the isolation results. Additionally, batch-to-batch variability of antibodies is also the key factor to affect reproducibility results. Currently, there is no universally accepted standard for MB-based EVs isolation due to the different beads recipes and isolation protocols, making it difficult to compare results across studies. Therefore, the development of standardized protocols, reference materials, and quality control measures is essential to improve reproducibility. Key criteria must include capture yield (e.g., % recovery of spiked EVs), purity (e.g., ratio of target EVs to co-isolated proteins), and specificity (e.g., enrichment of subpopulations in complex samples). Establishing standardized protocols and reference materials will enable objective comparison and reproducibility across studies and platforms. To conclude, reproducibility in EVs isolation is hindered by protocol variations and material inconsistencies. Establishing standardized methods and reference materials with defined metrics like yield, and purity.

### 6.3 EVs elution and integrity preservation

Recovering intact EVs after MB isolation is another challenge, because the elution conditions (e.g., low pH, detergents or enzymatic treatments) can damage EVs membranes and their biological functionality. Ideally, elution methods should efficiently release EVs while maintain their structural integrity and composition. The beads would interfere with downstream analyses, such as RNA sequencing or protein analysis. If the MBs are not entirely removed, they are likely to co-purify with EVs and lead to false positives or other artifacts (He et al., 2016). Developing mild elution approaches, such as reversible binding chemistries or cleavable linkers, is crucial to overcoming this limitation. Here we propose some potential solutions. Firstly, design stimuli-responsive systems. For instance, engineer the MBs with photothermal or electrochemical materials to enable on-demand release *via* external triggers such as near-infrared light or applied potential, inducing localized phase transitions or electrostatic repulsion. Secondly, the integration of cleavable linkers, such as reducible disulfide bonds (-S-S-) or photolabile moieties (e.g., o-nitrobenzyl), between MBs and capture ligands enables controlled EV release. Disulfide cleavage requires reducing agents (e.g., DTT, TCEP), while photocleavage offers reagent-free elution *via* specific wavelength exposure. Both strategies facilitate efficient recovery but necessitate post-elution purification to eliminate chemical or light-induced artifacts. In conclusion, eluting intact EVs without damage remains challenging due to harsh conditions that compromise membrane integrity and functionality. Solutions include developing stimuli-responsive release systems (e.g., photothermal/electrochemical triggers) and incorporating cleavable linkers (e.g., disulfide or photocleavable bonds), though these require post-elution purification to avoid interference in downstream assays.

## 7 Conclusion

This review introduces EVs and their importance in medicine while summarizing the conventional EVs isolation methods. Since the limitation in efficiency for these conventional size-based and charge-based isolation methods, the MBs-based isolation method and its isolation principle are proposed, as well as the application platform for MBs-based isolation. Moreover, the optimizations for increasing the isolation efficiency and challenges faced by MBs-based EVs isolation are also discussed. EVs and MBs are both appealing research fields in recent years in medicine. Each of the two possesses great potential in applications in medicine like detection, drug delivery, diagnosis, and so on. By integrating the two key hotspots into various MB-based platforms for EVs isolation, significant advancements can be achieved in enhancing isolation efficiency. This approach provides a robust foundation for subsequent applications in medical detection and diagnostics.
